# The effect of COVID-19 on emergency medical service call volumes and patient acuity: a cross-sectional study in Niagara, Ontario

**DOI:** 10.1186/s12873-021-00431-5

**Published:** 2021-03-29

**Authors:** Richard Ferron, Gina Agarwal, Rhiannon Cooper, Douglas Munkley

**Affiliations:** 1 Niagara Emergency Medical Service, Niagara, Canada; 2 Department of Family Medicine and Health Research Methods, Evidence and Impact, McMaster University, Hamilton, Canada

**Keywords:** Emergency medicine, Emergency medical services, COVID-19, Health services

## Abstract

**Background:**

The COVID-19 pandemic is a major public health problem. Subsequently, emergency medical services (EMS) have anecdotally experienced fluctuations in demand, with reports across Canada of both increased and decreased demand. Our primary objective was to assess the effect of the COVID-19 pandemic on call volumes for several determinants in Niagara Region EMS. Our secondary objective was to assess changes in paramedic-assigned patient acuity scores as determined using the Canadian Triage and Acuity Scale (CTAS).

**Methods:**

We analyzed data from a regional EMS database related to call type, volume, and patient acuity for January to May 2016–2020. We used statistical methods to assess differences in EMS calls between 2016 and 2019 and 2020.

**Results:**

A total of 114,507 EMS calls were made for the period of January 1 to May 26 between 2016 and 2020, inclusive. Overall, the incidence rate of EMS calls significantly decreased in 2020 compared to the total EMS calls in 2016–2019. Motor vehicle collisions decreased in 2020 relative to 2016–2019 (17%), while overdoses relatively increased (70%) in 2020 compared to 2016–2019. Calls for patients assigned a higher acuity score increased (CTAS 1) (4.1% vs. 2.9%).

**Conclusion:**

We confirmed that overall, EMS calls have decreased since the emergence of COVID-19. However, this effect on call volume was not consistent across all call determinants, as some call types rose while others decreased. These findings indicate that COVID-19 may have led to actual changes in emergency medical service demand and will be of interest to other services planning for future pandemics or further waves of COVID-19.

## Background

The global outbreak of severe acute respiratory syndrome coronavirus 2 (SARS-CoV-2), the virus that causes coronavirus disease 2019 (COVID-19), is a major public health problem. The first case of COVID-19 in Canada was documented on January 25th, 2020 in Ontario [1]. Since then, Ontario has seen a large COVID-19 outbreak and at the time of writing is continuing to experience a rise in the number of positive cases [1]. As the impact of the COVID-19 pandemic on health care systems has become a concern in Ontario, paramedic services have begun to plan for anticipated impacts. Planning of this nature involves the ongoing monitoring of call volumes and types to anticipate future resource needs. There has been mixed information concerning the demand for emergency medical services (EMS). Perceptions abound that EMS will experience potential surges, during which containment is challenging yet crucial to the success of the health system response [2, 3]. These are countered by anecdotes that EMS calls have decreased, as people are avoiding involvement with the health system for fear of infection [4].

Volumes of EMS calls are important to understand since they impact the response times, and are part of quality of EMS care [5]. Fluctuations in call volumes can result in more or less EMS resources available to attend to emergencies and could affect patient outcomes [6]. Therefore, in order to adequately plan and assist other EMS in Ontario and across Canada with similar planning, problems, and questions, it is important to understand the precise effect of the pandemic on EMS. Niagara EMS (NEMS) provides emergency medical services for an area in Southern Ontario with a population of about half a million people [7]. The average age of the population is 44 years old, with 64% of the population falling into the category of being 15 to 64 years of age; 48% are male; 89% are of European descent; 4% are Indigenous peoples; between 1 and 11% describe themselves as having non-European heritage (e.g. Asian); median post-tax family income is $72,105 and single-parent family income is $46,684 [7].

NEMS is a large service that, in June of 2016, had approximately 3.9% of Ontario’s EMS call volumes [8]. Niagara region operates both an ambulance communications service with a dispatch centre, and a land ambulance service providing primary and advanced care paramedic services. There are approximately 343 full and part time paramedics in employment with the service, and 33 ambulances staffed at peak. These characteristics allow for reasonable comparisons to be made with other EMS service in Ontario. Though EMS in Ontario appears to have been affected by the COVID-19 pandemic, the specific impact of COVID-19 on Ontario EMS has not yet been critically and robustly examined. The following is an assessment of the change in call types and acuity before and during the first wave of COVID-19.

## Methods

### Study design

A cross-sectional analysis of de-identified data obtained from the NEMS database (EDGE) was conducted. Electronic Patient Care Record (ePCR) data for each patient was extracted from the database. A time period of January 1 to  May 26, 2020 only was chosen to reflect the onset and establishment phases of the pandemic. Further data from the same time period from 2016 to 2019 were extracted. These time periods were chosen to be comparable due to seasonal variation in EMS calls at the daily, monthly, and yearly level [9]. The aim of this study was to examine the impact of the ongoing COVID-19 pandemic on EMS call volumes, determinants (types of calls), and patient acuity at scene of arrival. Research ethics board approval was sought from the Hamilton Integrated Research Ethics Board, a partnership of McMaster University, St. Joseph’s Healthcare Hamilton, and Hamilton Health Sciences; the project was deemed not to require ethics review because it was a quality improvement project using aggregate data, and an exemption was granted.

### Setting

NEMS serves 12 municipalities and 1850 km^2^ that comprise the Niagara Region of Ontario [10]. As of the 2016 census, the population served by NEMS consisted of 447,888 people [7].

### Data collation

The NEMS ambulance communications center utilizes the Medical Priority Dispatch System (MPDS®) to categorize and triage calls coming into the center. Paramedic service data from both land ambulance and dispatch were accessed through a business intelligence solutions product called SAP Business Objects Edge Edition’ EDGE [11]. Information regarding call type was extracted from the computer aided dispatch (CAD) software, in addition to the ePCR data pertaining to each patient.

### Measures

EMS calls were reported as counts and were stratified by call determinant type (e.g. abdominal pain). We examined changes in patient acuity levels from January 1 2019 to May 26 2020. Patient acuity was measured through the Canadian Triage and Acuity Scale (CTAS), a validated 5-level triage score used by paramedics on scene arrival [12]. Level 1 indicates a highly urgent situation while level 5 indicates a non-urgent situation.

### Data analysis

The total number of EMS calls were tabulated for each determinant and year, and then summed for the time period of 2016–2019. Chi-square tests of independence were conducted to compare the proportion of EMS calls in 2020 to EMS calls in 2016–2019 for each call determinant. Fisher’s exact test was used for determinants with frequencies of 5 EMS calls or less. We also conducted a chi-square test of independence to compare the incidence rate ratios of EMS calls in 2020 to 2016–2019. Using population data for 2016, 2017, and 2019 in the Niagara region estimated by Statistics Canada [13], we made our own estimate of the population growth rate to calculate the projected population of Niagara in 2018 and 2020 using the following formulas:

$$ k=\frac{P_t-{P}_{t_o}}{P_{t_o}\ast \left(t-{t}_o\right)} $$

where k is the population growth rate, P_t_ is the population size in 2019, ${P}_{t_o}$ is the population size in 2017, and t and t_o_ represent 2019 and 2017, respectively.

$$ {P}_t={P}_o{e}^{k\left(t-{t}_o\right)} $$

where ${P}_{t_o}$ is the population of Niagara in 2017, k is the population growth rate, and t and t_o_ represent 2020 (and 2018) and 2017, respectively.

We used these projections to calculate the incidence rate ratios of EMS calls in 2016–2019 and in 2020, with person-years as the denominator and number of EMS calls as the numerator.

Changes in patient acuity were assessed for the timeframe of January 1^st^, 2019 to May 26^th^, 2020 using chi-square tests of independence to compare total calls in 2016–2019 to the total calls in 2020.

Data were analyzed in RStudio desktop version 1.2.50331 using R version 3.6.1 [14].

## Results

A total of 114,507 EMS calls were made to NEMS for the period of January 1 to May 26 between 2016 and 2020, inclusive (Table 1). Of the total calls from 2016 to 2020, determinants representing a larger absolute proportion of all calls include sick person (16.8%), falls (15.4%), breathing problems (11.2%), and chest pain (8.9%). Overall, the incidence rate of EMS calls per person-year decreased in 2020 compared to 2016–2019.

**Table 1:** Five Year comparison of incidents and call types, January–May 2016–2020

EMS Call Type and Determinant Number	EMS Calls 2016 n (%)	EMS Calls 2017 n (%)	EMS Calls 2018 n (%)	EMS Calls 2019 n (%)	EMS Calls 2020 n (%)	4-Year Average of EMS Calls (2016–2019)	Proportion of calls in 2020 vs. calls 2016–2019	Relative Change in Calls (2020 vs. 2016–2019)	Chi Square Test of Independence (Chi-score, ***p***-value)
Abdominal Pain	784 (3.7)	784 (3.5)	806 (3.3)	885 (3.6)	808 (3.6)	814.75 (3.5)	3.8% vs. 3.7%	3%	*χ*^2^ (1) = 0.59 *p* = 0.44
Allergies	167 (0.8)	173 (0.8)	174 (0.7)	175 (0.7)	159 (0.7)	172.25 (0.8)	0.7% vs. 0.8%	−4%	*χ*^2^ (1) = 0.23 *p* = 0.63
Animal Bites/Attacks	24 (0.1)	24 (0.1)	27 (0.1)	22 (0.1)	32 (0.1)	24.25 (0.1)	0.1% vs. 0.1%	37%	*χ*^2^ (1) = 2.42 *p* = 0.12
Assault/ Sexual Assault	695 (3.3)	710 (3.1)	671 (2.8)	629 (2.6)	682 (3.1)	676.25 (2.9)	3.2% vs. 3.0%	4%	*χ*^2^ (1) = 1.22 *p* = 0.27
Back Pain	231 (1.1)	352 (1.6)	370 (1.5)	367 (1.5)	304 (1.4)	330 (1.4)	1.4% vs. 1.5%	-4%	*χ*^2^ (1) = 0.49 *p* = 0.47
Breathing Problems	2297 (10.9)	2385 (10.6)	2521 (10.4)	2310 (9.5)	2052 (9.2)	2378.25 (10.3)	10.2% vs. 11.5%	−12%	*χ*^2^ (1) = 22.53 ***p*** < 0.01*
Burns/ Explosions	37 (0.2)	35 (0.2)	62 (0.3)	72 (0.3)	51 (0.2)	51.5 (0.2)	0.2% vs. 0.2%	4%	*χ*^2^ (1) = 0.033 *p* = 0.86
Carbon Monoxide/ HAZMAT	65 (0.3)	67 (0.3)	78 (0.3)	75 (0.3)	47 (0.2)	71.25 (0.3)	0.2% vs. 0.3%	−31%	*χ*^2^ (1) = 5.85 ***p*** = 0.016*
Cardiac Arrest	255 (1.2)	321 (1.4)	369 (1.5)	336 (1.4)	385 (1.7)	320.25 (1.4)	1.8% vs. 1.4%	26%	*χ*^2^ (1) = 14.89 ***p*** < 0.01*
Chest Pain	1798 (8.5)	1765 (7.8)	2076 (8.6)	2007 (8.2)	1790 (8.1)	1911.5 (8.3)	8.8% vs. 9.0%	−3%	*χ*^2^ (1) = 1.21 *p* = 0.27
Choking	58 (0.3)	76 (0.3)	78 (0.3)	104 (0.4)	54 (0.2)	79 (0.3)	0.2% vs. 0.3%	−29%	*χ*^2^ (1) = 5.48 ***p*** = 0.019*
Convulsions	731 (3.5)	784 (3.5)	778 (3.2)	814 (3.3)	730 (3.3)	776.75 (3.4)	3.4% vs. 3.5%	−3%	*χ*^2^ (1) = 0.35 *p* = 0.55
Diabetic	278 (1.3)	268 (1.2)	268 (1.1)	249 (1.0)	203 (0.9)	265.75 (1.2)	0.9% vs. 1.2%	−21%	*χ*^2^ (1) = 9.26 ***p*** < 0.01*
Drowning/ Diving Accident	11 (0.1)	< 5	8 (0.0)	9 (0.0)	6 (0.0)	8 (0.0)	0.0% vs.0 .0%	−23%	*p* = 0.69
Electrocution/Lightning	5 (0.0)	< 5	< 5	< 5	< 5	< 5	0.0% vs. 0.0%	−74%	*p* = 0.22
Eye Problems/ Injuries	27 (0.1)	24 (0.1)	21 (0.1)	20 (0.1)	23 (0.1)	23 (0.1)	0.1% vs. 0.1%	3%	*χ*^2^ (1) = 0.027 *p* = 0.87
Falls	2715 (12.9)	3050 (13.5)	3277 (13.5)	3248 (13.3)	3001 (13.5)	3072.5 (13.3)	15.6% vs. 15.4%	2%	*χ*^2^ (1) = 0.59 *p* = 0.44
Headache	145 (0.7)	127 (0.6)	115 (0.5)	155 (0.6)	146 (0.7)	135.5 (0.6)	0.7% vs. 0.6%	11%	*χ*^2^ (1) = 1.47 *p* = 0.23
Heart Problems	395 (1.9)	314 (1.4)	370 (1.5)	424 (1.7)	423 (1.9)	375.75 (1.6)	1.9% vs. 1.7%	17%	*χ*^2^ (1) = 8.24 ***p*** = 0.0041*
Heat/Cold Exposure	19 (0.1)	19 (0.1)	30 (0.1)	40 (0.2)	34 (0.2)	27 (0.1)	0.2% vs. 0.1%	33%	*χ*^2^ (1) = 1.88 *p* = 0.17
Hemorrhage/ Laceration	790 (3.8)	859 (3.8)	946 (3.9)	898 (3.7)	826 (3.7)	873.25 (3.8)	3.9% vs. 3.9%	−2%	*χ*^2^ (1) = 0.21 *p* = 0.64
Inaccessible Incident	< 5	< 5	< 5	< 5	5 (0.0)	2.75 (0.0)	0.0% vs. 0.0%	90%	*p* = 0.22
Overdose/ Poisoning	416 (1.9)	503 (2.2)	548 (2.3)	865 (3.5)	928 (4.2)	583 (2.3)	4.4% vs. 2.6%	70%	*χ*^2^ (1) = 176.49 ***p*** < 0.01*
Pregnancy	83 (0.4)	90 (0.4)	90 (0.4)	108 (0.4)	105 (0.5)	92.75 (0.4)	0.5% vs. 0.4%	18%	*χ*^2^ (1) = 11.80 ***p <*** 0.01*
Psychiatric/ Abnormal Behaviour	1284 (6.1)	1467 (6.5)	1529 (6.3)	1588 (6.5)	1426 (6.4)	1467 (6.4)	6.9% vs. 6.8%	1%	*χ*^2^ (1) = 0.12 *p* = 0.73
Sick Person	3362 (15.9)	3589 (15.9)	3797 (15.7)	3495 (14.3)	2255 (10.2)	3560.75 (15.4)	11.3% vs. 18.2%	−38%	*χ*^2^ (1) = 404.71 ***p*** < 0.01*
Stab/Gunshot	34 (0.2)	27 (0.1)	27 (0.1)	36 (0.2)	42 (0.2)	31 (0.1)	0.2% vs. 0.1%	40%	*χ*^2^ (1) = 3.71 *p* = 0.054
Stroke	619 (2.9)	635 (2.8)	707 (2.9)	690 (2.8)	717 (3.2)	662.75 (2.9)	3.3% vs. 3.%	13%	*χ*^2^ (1) = 7.93 ***p*** < 0.01*
Motor Vehicle Collision	788 (3.7)	837 (3.7)	881 (3.6)	903 (3.7)	682 (3.1)	852.25 (3.7)	3.2% vs. 3.8%	−17%	*χ*^2^ (1) = 20.18 ***p*** < 0.01*
Traumatic Injuries	500 (2.4)	484 (2.1)	484 (2.0)	487 (1.9)	403 (1.8)	488.75 (2.1)	1.8% vs. 2.2%	−15%	*χ*^2^ (1) = 8.20 ***p*** = 0.0042*
Unconscious	1541 (7.3)	1689 (7.5)	2018 (8.3)	2033 (8.3)	1960 (8.8)	1820.25 (7.9)	9.7% vs. 8.6%	13%	*χ*^2^ (1) = 21.11 ***p*** < 0.01*
Unknown Problem	902 (4.3)	1111 (4.9)	1105 (4.6)	1369 (5.6)	872 (3.9)	1121.75 (4.9)	4.1% vs. 5.1%	−20%	*χ*^2^ (1) = 35.14 ***p*** < 0.01*
Pandemic Protocol	0	0	0	0	1060 (4.8)	0	5.0% vs. 0.0%	3%	***p*** < 0.01*

*Indicates significance

### Call volumes and type

Several types of EMS calls increased during January–May 2020 when compared to the same time frame in 2016–2019. Niagara had its first COVID-19 case on March 13^th^, 2020; therefore, EMS call volumes were most likely minimally affected in January and February. EMS calls significantly increased (*p* < 0.01) for cardiac arrest (1.8% versus 1.4%), heart problems (1.9% versus 1.7%), overdose/poisoning (4.4% versus 2.6%), pregnancy (0.5% versus 0.4%), stroke (3.3% versus 2.9%), and unconscious (9.7% versus 8.6%); see Table 1.

However, compared to 2016–2019, EMS calls significantly decreased (*p* < 0.05) in 2020 for breathing problems (10.2% versus 11.5%), carbon monoxide/HAZMAT (0.2% versus 0.3%), choking (0.2% versus 0.3%), chest pain (8.8% versus 9.0%), diabetic (0.9% versus 1.2%), sick person (11.3% versus 18.3%), motor vehicle collision (3.2% versus 3.8%), and unknown problem (4.1% versus 5.1%).

The incidence rate of EMS calls in Niagara 2020 was significantly lower (*p* < 0.01) than EMS calls for the same time-period in 2016–2019 (0.046 versus 0.049); see Table 2.

**Table 2:** Comparison of total calls per person-year in 2016–2019 to 2020

	2016	2017	2018	2019	2020	Average EMS calls per person-year 2016–2019	Chi Square Test of Independence (Chi-score, *p*-value)
**EMS calls per person-year**	0.047	0.048	0.051	0.051	0.046	0.049	*χ*^2^ (1) = 75.66 ***p*** < 0.01*
	**2016**	**2017**	**2018**	**2019**	**2020**		
**Population**	**447,888**	**465,569**	**472,416**	**479,183**	**486,414**		

*Indicates significance Population values for 2018 and 2020 were projected using the formula written in the methods section

### Patient acuity

EMS calls significantly increased (*p* < 0.01) for calls assigned a CTAS level of 1 (4.0% versus 2.9%) and for CTAS level 4 (14.3% versus 12.3%); see Table 3. EMS calls significantly decreased (*p* < 0.01) for calls assigned a CTAS level of 2 (22.9% versus 24.3%) and CTAS level 3 (78.4% versus 94.1%).

**Table 3:** Five Year comparison of CTAS Scores, January–May 2016–2020

CTAS Level	Total EMS Calls in 2016 n (%)	Total EMS Calls in 2017 n (%)	Total EMS Calls in 2018 n (%)	Total EMS Calls in 2019 n (%)	Total EMS Calls in 2020 n (%)	Total Calls for Each Level	Proportion of calls in 2020 vs. calls 2016–2019	Relative Change in Calls	Chi Square Test of Independence (Chi-score, ***p***-value)
1 (Resuscitation)	515 (2.4)	605 (2.7)	749 (3.0)	773 (3.1)	894 (3.9)	3536 (3.1)	4.1% vs. 2.9%	−41%	*χ*^2^ (1) = 70.36 ***p*** < 0.01*
2 (Emergent)	4509 (21.4)	4491 (20.0)	4656 (18.8)	4550 (18.4)	4261 (18.6)	22,467 (19.4)	22.9% vs. 24.3%	6%	*χ*^2^ (1) = 10.90 ***p*** = 0.01*
3 (Urgent)	10,996 (52.1)	11,137 (49.7)	11,513 (46.5)	11,457 (46.3)	10,060 (43.9)	55,163 (47.6)	78.4% vs. 94.1%	10%	*χ*^2^ (1) = 152.61 ***p*** < 0.01*
4 (Less Urgent)	1935 (9.2)	2325 (10.4)	2953 (11.9)	2954 (11.9)	2856 (12.5)	13,023 (11.2)	14.3% vs. 12.3%	−17%	*χ*^2^ (1) = 43.89 ***p*** < 0.01*
5 (Non-Urgent)	2091 (9.9)	2453 (10.9)	3185 (12.9)	3181 (12.8)	2650(11.6)	13,560 (11.7)	13.1% vs. 13.3%	−1%	*χ*^2^ (1) = 0.069 *p* = 0.79

*Indicates significance. Proportions were calculated using total calls and calls not assigned a CTAS number were not included in the table

## Discussion

In our study we found that overall, the incidence rate of EMS calls from Jan-May 2020 significantly decreased compared to EMS calls made during the same timeframe in 2016–2019. This is in keeping with anecdotal reports in Canada describing a decrease in EMS calls beginning in April 2020 [4, 15]. In the U.S, a survey found that people were abstaining from calling 9-1-1, regardless of the severity of the health event, due to fear of COVID-19 [16]. A serious consequence of this behaviour of avoiding the ED is that high acuity patients are waiting to seek necessary care until they are too unwell to be treated adequately [17].

We found that call volumes for several determinants increased, such as 9-1-1 calls for cardiac arrest, stroke, and heart attacks. Evidence exists in the literature to support the finding of increased cardiac arrests. Data from Northern Italy shows that during the pandemic, out-of-hospital cardiac arrests has increased by 58.0% [18]. A New York study has found that the incidence of out-of-hospital cardiac arrests needing resuscitation has tripled in 2020 compared to the same time frames in 2019 [19]. Other US data shows that 911 calls for cardiac arrest rose in March by 45.0%, likely because people have been waiting too long before seeking care [17]. Indeed, fear of contracting COVID-19 may have contributed to EMS avoidance, even for patients experiencing life-threatening health events, resulting in the apparent increase in cardiac events and stroke. There is some evidence to suggest that our findings of increased cardiac arrests could also be related to SARS-CoV-2 itself. A systematic review found a correlation between COVID-19 and cardiovascular complications, despite COVID-19 being a respiratory disease [20]. However, we were not able to examine if any of the patients presenting with cardiac arrest or heart attack were diagnosed with COVID-19.

In addition to negatively impacting physical health, the COVID-19 pandemic has also been a threat to mental health [21, 22]. Overdose/poisoning demonstrated the largest increase in call volumes than any other call determinant. One group at an elevated risk for experiencing the negative mental health impacts of the COVID-19 pandemic are those who used substances or are in recovery [23]. In order to control the spread of COVID-19, multiple safe injection sites have remained closed during the pandemic. It is possible that these closures could have contributed to the alarming increase in overdoses/poisonings seen in the current study. Some safe injection sites in Ontario have reopened after noticing an increase in overdoses and noting the role that these sites play in harm reduction [24]. The interruption of regular primary health care and the switch to use of virtual care versus in-person care may also have impacted overdose numbers. Patients using prescription opioids may be monitored by urine testing less frequently and primary care may have been forced to be less vigilant while prescribing narcotics in these altered circumstances. Additionally, physical distancing measures may have led to an increase in social isolation and stress, increasing the risk of overdose for those who use substances due to lack of support and emotional strain. In support of this theory, lack of social support has been shown to predict non-fatal drug overdose in females, and mental health problems, such as depression, have been shown to predict overdose in both males and females [25]. It is worth noting that 9-1-1 calls for mental health appear to have decreased during the pandemic, although not significantly. Previously, 9-1-1 may have been called prior to an overdose, and those cases would have been identified as mental health issues. However, if patients are avoiding calling 9–1-1, there may be a subsequent increase in incidences of overdose.

Several call determinants decreased from January 2020 to May 2020. Unexpectedly, EMS calls regarding breathing problems and sick people decreased. An explanation for this could be the introduction of a new MPDS card on April 30, 2020; the ‘pandemic protocol card’. Calls that would have previously been categorized as “sick person” or “breathing problems”, the determinants associated with COVID-19, became categorized as ‘pandemic protocol’. The pandemic protocol card was meant to provide an opportunity for an altered response to call types identified as potential COVID-19 cases during periods that threaten to overwhelm EMS resources. The protocol encompasses a variety of presentations, including shortness of breath and chest pain. For this reason, categorizing a patient in only one protocol would result in misclassification. Therefore, call volumes for breathing problems and sick person may not have decreased as much as we reported.

Of all determinants that experienced a decrease in call volumes, motor vehicle collisions (MVC) decreased the most. People may have been commuting and travelling less frequently and had been encouraged to stay at home, therefore were less likely to become involved in car accidents. A study in Michigan, USA, examining the effect of shelter-in-place orders on orthopaedic trauma, also found that motor vehicle collisions decreased by 17% in March 2020 relative to March 2019 [26]. Although Ontario did not issue a shelter-in-place order, citizens were instructed to stay-at-home which may offer an explanation for the decrease in the number of MVC.

The number of patients presenting as high acuity (CTAS Level 1) and lower acuity (CTAS level 4) increased during the pandemic. A possible explanation for this is that lower acuity patients are not seeking care in time, so that when EMS is called they present as high acuity [16]. However, the increase in overdoses could also be contributing to higher patient acuity upon scene arrival. EMS calls for emergent (CTAS Level 2) and urgent (CTAS Level 3) situations have decreased from January 2019 to May 2020. This finding may also be explained by a fear of contracting COVID-19 in emergency rooms, and therefore avoiding EMS.

### Limitations

We cannot solely attribute the decrease in EMS calls to the COVID-19 pandemic. EMS calls could also have decreased due to changes at dispatch because of an MPDS system upgrade that was implemented in 2019. Additionally, in the same year the triage process was changed for certain conditions, a Mobile Integrated Health Team, with additional mental health training, may have been dispatched instead of a regular ambulance. The decrease in EMS calls may also be attributed to the lack of tourists in attendance in the region, which typically accounts for a portion of the Niagara EMS calls each year. The population of Niagara has 13 million visitors each year, and a decrease in visitors may lead to a decrease in 9-1-1 calls [27]. Though the current data were not collated on an urban vs. rural basis, it is likely that the drop in EMS calls is driven by urban regions, where tourism is high in the spring and summer. In addition, the rules for the classification of call determinants change every 1–2 years. However, we have confirmed that call determinants have mostly stayed the same, and any changes that were made would have not affected the selection of determinants chosen at the time of dispatch. There may also be variation between the call determinant assigned at the time of triage, based on the assessment of the paramedics. With regards to patient acuity, we cannot rule out the possibility that paramedic documented CTAS may not be completely accurate as it is a subjective measure.

## Conclusion

We have confirmed that overall, EMS calls in the Niagara region have significantly decreased during the first 5 months of the COVID-19 pandemic, whilst some types increased. Specifically, EMS calls for overdose increased the most out of any other call determinant, while calls for motor vehicle collisions decreased more than any other determinant. Calls related to high patient acuity levels significantly increased, while EMS calls for urgent and emergent patients significantly decreased. Our findings suggest that the COVID-19 pandemic could have contributed to a decrease in EMS calls overall, and increased certain types of calls, including an increase in the number of high acuity patients. The results of this study may be generalizable to other geographical areas with similar population sizes and characteristics but may not be generalizable to areas that don’t provide universal healthcare. These findings may be of interest to other EMS as they plan for future pandemics or second waves of COVID-19, including the ability to predict call type in an effort to adjust a targeted response. This information can also provide an opportunity for dialogue between EMS agencies and health care partners on the consequences of delays in seeking treatment.

## Data Availability

The data that support the findings of this study are available from Niagara Emergency Medical Services, but restrictions apply to the availability of these data, which were used under license for the current study, and so are not publicly available. Data are however available from the authors upon reasonable request and with permission of Rick Ferron.

## Abbreviations

SARS-CoV-2: severe acute respiratory syndrome coronavirus 2
COVID-19: coronavirus disease 2019
EMS: Emergency medical services
NEMS: Niagara emergency medical service
ePCR: electronic Patient Care Record
MPDS®: Medical priority dispatch system
CAD: Computer aided dispatch
CTAS: Canadian triage and acuity scale
MVC: Motor vehicle collisions
